# A Pilot Trial of Stepwise Implementation of Virtual Reality Mindfulness and Accelerated Transcranial Magnetic Stimulation Treatments for Dysphoria in Neuropsychiatric Disorders

**DOI:** 10.1155/2023/9025984

**Published:** 2023-11-22

**Authors:** Megan C. Senda, Kevin A. Johnson, Isabelle M. Taylor, Mariah M. Jensen, Yang Hou, F. Andrew Kozel

**Affiliations:** Department of Behavioral Sciences and Social Medicine, Florida State University College of Medicine, Florida State University, Tallahassee, FL, USA

## Abstract

Dysphoria is a transdiagnostic symptom that causes considerable suffering. Implementation of established self-care and clinical treatment options, such as mindfulness and transcranial magnetic stimulation (TMS), is typically disjointed for conditions involving dysphoria. There is a need for a rapid progression of accessible treatments that can be efficacious across multiple comorbidities. In a pilot stepwise implementation study to assess feasibility and effectiveness, adult participants with dysphoria (depression, anxiety, PTSD, and/or chronic pain) went through a treatment course of VR mindfulness, then accelerated TMS (accel-TMS) over the left dorsolateral prefrontal cortex (left dlPFC), then accel-TMS over the dorsomedial prefrontal cortex (dmPFC). Participants who did not benefit from one treatment phase progressed to the next until remission or study completion. Twenty-four participants were enrolled with 23 in VR mindfulness (phase 1), 19 in accel-TMS left dlPFC (phase 2A), and 13 in accel-TMS dmPFC (phase 2B). For our primary outcome measure of the short form-36 emotional well-being subscale (paired *t*-test), no significant change was found in phase 1 (*n* = 19, *p* = .226), significant improvement was found in phase 2A (*n* = 19, *p* = .038), and no significant change was found in the smaller sample of phase 2B (*n* = 12, *p* = .089). Symptom improvement was largely supported by clinician-administered scales, with more significant changes found in accel-TMS left dlPFC and dmPFC. The benefits of VR mindfulness were limited; however, both accel-TMS phases showed a significant impact on secondary measures of depression, anxiety, and PTSD. This stepwise protocol shows promise in providing an approach to rapidly improve symptoms of dysphoria in transdiagnostic populations. This trial is registered with NCT05061745.

## 1. Introduction

Treatment response within a specific diagnosis of a neuropsychiatric disorder is highly variable due to individual heterogeneity, and many treatments have efficacy across multiple diagnoses (often referred to as comorbid diagnoses) [1, 2]. We sought to identify a transdiagnostic concept that would capture a prevalent and chief concern across a broad clinical population within the context of a related subset of psychiatric disorders. Dysphoria is a dictionary-defined term describing feelings of unease, unhappiness, or dissatisfaction that characterizes the central symptoms across a range of diagnoses including mood, anxiety, trauma, and chronic pain conditions [3]. There is no treatment with demonstrated efficacy and tolerability specifically for the transdiagnostic condition of dysphoria. There is a need, however, to develop treatment approaches that will address dysphoria in patients [4]. Dysphoria is not an official clinical or research diagnostic criterion; however, we attempted to define and treat a broad cohort who shared overlapping symptomology associated with mood, anxiety, trauma, and chronic pain disorders. Mindfulness and transcranial magnetic stimulation (TMS) are both established therapies deployed separately for various disorders, with recent advances in delivery that increase the potential utility across multiple conditions involving dysphoria.

Mindfulness has reported benefits for depression, anxiety, PTSD, and pain; however, its broad implementation has yet to be fully optimized [5, 6]. Traditionally, patients are trained in mindfulness through group or individual instruction, and environments free of distractions are conducive to mindfulness exercises. Virtual reality (VR) is a powerful tool to deliver mindfulness exercises as well as other types of therapies [7–9]. Unlike other media formats, the sensory immersion of VR supports strong dissociation from environmental factors to guide focus on the exercises. Additionally, device-based interventions remove the resource and cost burden of in-person instruction. As the first step, we sought to gauge the feasibility and effectiveness of VR mindfulness as a treatment for dysphoria.

TMS is a noninvasive method for stimulating the brain, with FDA-cleared indications for major depressive disorder, “anxious depression,” obsessive-compulsive disorder, and smoking cessation. There is also building evidence for efficacy in PTSD and some chronic pain conditions [10, 11]. “Accelerated TMS” is a potential major advancement in the field [12]. The standard protocol of TMS for depression involves one daily session distributed over six weeks [13, 14], while accelerated protocols use multiple treatments per day in an attempt to accelerate response. Some initial studies examining the increased number of TMS treatments per day failed to provide impressive results compared to standard TMS treatment regimens [15]. More recent studies, however, with an increase in the number of sessions per day, have shown remarkable results. These accelerated protocols involved 5-10 treatment sessions per day and have demonstrated symptom improvement in days rather than weeks [16–18]. This rapid improvement of accelerated protocols has the potential to allow for faster determination of efficacy, so that patients can advance to an adjusted protocol (e.g., different brain location) or different therapy in case of inadequate clinical response. In addition, accelerated protocols can make TMS more accessible, as the logistics of a week's break from life demands (e.g., work, school, or childcare) may be less disruptive than daily visits for six weeks (especially for those not near a treatment clinic).

The goal of this project was to pilot a study to examine the implementation and feasibility, as well as preliminarily assess the effectiveness, of VR Mindfulness and two variants of accelerated TMS (accel-TMS) in a stepwise manner to rapidly evaluate multiple approaches for treating dysphoria. Participants would only progress to the next phase if their symptoms of dysphoria were not resolved. In the first phase (phase 1: VR mindfulness), we sought to determine the feasibility, tolerability, and effectiveness of mindfulness delivered through VR for treating depression, anxiety, PTSD, and pain symptoms. We hypothesized that it would be feasible to administer VR technology for dysphoria, as well as significantly improve dysphoria across the entire sample and within specific diagnostic groups. In the second phase (phase 2: accel-TMS), we sought to demonstrate the feasibility, tolerability, and effectiveness of accel-TMS for dysphoria starting with sessions stimulating the left dorsolateral prefrontal cortex (left dlPFC) (phase 2A). Those with inadequate treatment response to phase 2A were treated by stimulating the dorsomedial prefrontal cortex (dmPFC) (phase 2B). We hypothesized that accel-TMS would be well-tolerated and feasible, as well as significantly improve dysphoria across the entire sample and within specific diagnostic groups. We plan to use the results of this study to guide larger trials aimed at improving the implementation and accessibility of tools for managing dysphoria.

## 2. Materials and Methods

### 2.1. Brief Overview

**Figure 1:**
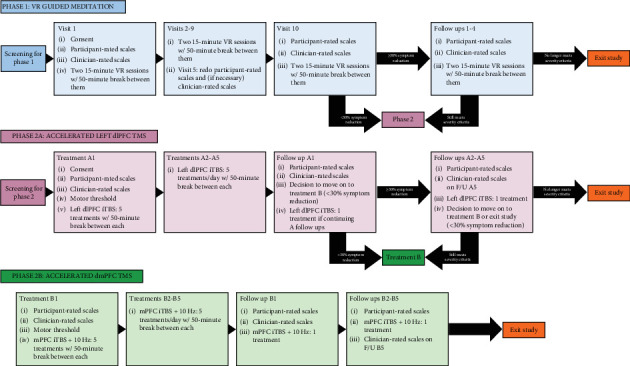
Study overview.

Figure 1 provides a visual overview of the study structure to aid in following the study flow. Our stepwise approach allowed participants to advance to the next treatment if there was the potential for future benefit (as indicated by self-report measures). The SF-36 emotional well-being subscale assessed benefits across the entire sample, further supported by diagnostic-specific scales for subgroups.

The Florida State University Investigation Review Board approved the study, and we registered the protocol on ClinicalTrials.gov (Neuromodulation for Dysphoria: NCT05061745) prior to any participants being enrolled. Participants consented verbally to be prescreened for eligibility and safety for the study by phone. We gathered written consent from participants at the beginning of each phase prior to any procedures being instituted. Participants were first enrolled in phase 1, involving two weeks of VR-guided mindfulness. Those that did not adequately respond were screened for phase 2A. Those who qualified and provided written consent moved on to phase 2A utilizing accel-TMS of the left dlPFC. Those that did not adequately respond to this phase moved to phase 2B which utilized accel-TMS of the dmPFC.

### 2.2. Inclusion/Exclusion Criteria

Inclusion criteria for this study included minimum symptoms of dysphoria based on self-rated scales (patient health questionnaire-9 (PHQ-9) ≥10; general anxiety disorder-7 (GAD-7) ≥10; PTSD checklist for DSM-5 (PCL-5) ≥45; or average pain (as assessed by patient-reported outcomes measurement information system (PROMIS) pain intensity short form 3a v2.0) ≥4/10 for >3 months), at least 18 years of age, and no changes in medication for at least one month. Exclusion criteria included taking any medications that increased the risk for TMS, diagnosis of a substance use disorder, neurocognitive disorder, severe neurological disorder, psychotic disorder, history of severe traumatic brain injury (TBI), metal within the head, current pregnancy, and unstable medical conditions. We used urine testing to screen for substance use and pregnancy.

### 2.3. Phase 1: VR Mindfulness

At baseline, participants completed a battery of rating scales (permission for use of the profile of mood states was given by the author, see Table 1). We performed a clinical interview to assess for psychiatric/pain disorders and determine the primary scale to assess symptoms and safety. Participants then trialed two daily VR sessions (Guided Meditation VR©, Cubicle Ninjas, Glen Ellyn, IN; Valve Index® Headset, Valve Corporation, Bellevue, WA) that lasted 15 minutes separated by a 50-minute break for ten days. The length of VR sessions and breaks were modeled to be similar to the accel-TMS sessions to acclimate the participants to the intensive schedule without initially overwhelming them (i.e., 2 sessions per day for VR versus five sessions per day for accel-TMS). Any side effects or problems with the VR sessions were noted. On visits 5 and 10, participants completed rating scales. Participants who did not experience wellness benefit from phase 1 or chose to progress to TMS were evaluated for phase 2. Wellness benefit, used to determine progression through the study, was defined as a ≥30% improvement on the clinician-rated scale that was identified by investigators to track the primary disorder (i.e., depression = Montgomery-Asperg depression rating scale (MADRS); anxiety = Hamilton anxiety rating scale (HAM-A); PTSD = clinician-administered PTSD scale for DSM-5 (CAPS-5); chronic pain = clinician-administered PROMIS pain interference short form 6b v1.1). As this was a pilot study, we did not know how participant's symptoms would progress over time. We wanted to balance giving time for the treatment effect of the accel-TMS protocol to fully manifest without unnecessarily delaying the progression to a potentially effective different treatment. The 30% threshold was chosen to balance these concerns based on recent studies examining predictors of TMS treatment outcomes at 1-2 weeks (5-10 treatments) during a standard treatment course (6 weeks of daily sessions) [19, 20]. As we were assessing after 5 days of 25 treatments, we chose a more stringent criteria (30% versus 20%) to determine progression to the next phase. Additionally, we sought to define meaningful change that was reasonable across the various measures of all conditions.

### 2.4. Phase 2: Accelerated TMS

Minimum symptom severity eligibility for continuation in the study was determined by the primary self-rated scales just as for phase 1. At treatment A1, participant- and clinician-rated scales were completed to assess for psychiatric/pain disorders and determine the primary scale to assess symptoms (determination of primary scale for a particular participant could be different from phase 1; see Table 1).

For the acute period of phase 2A (treatment days A1–A5, typically 5 contiguous days), there were five 10-minute TMS sessions (MagVenture, Inc. MagPro R30 stimulator, Denmark) per day with a 50-minute break between each session. We assessed for wellness benefit at follow-up A1 (about one week posttreatment) using the primary clinician-rated scale to assess symptoms. Those that achieved benefit were treated with TMS once and followed weekly (follow-ups A2–A5) for four additional visits (i.e., rating scales and one TMS treatment). Participants who failed to achieve “wellness benefit” at follow-up A1 exited phase 2A and had the option to start phase 2B. Participants could also continue to phase 2B if they still met any inclusion criteria for dysphoria at follow-up A5.

For the acute period of phase 2B (treatment days B1–B5, typically 5 contiguous days), there were five 10-minute TMS sessions per day with a 50-minute break between each session. For subsequent weekly visits (follow-ups B1–B5), there was only 1 session per day (i.e., rating scales and one TMS treatment). Clinician- and self-rated scales were administered on the same schedule as in phase 2A (Table 1).

#### 2.4.1. Treatment 2A Parameters (Accel-TMS iTBS Left dlPFC)

Stimulation was delivered with a cool B70 coil positioned over the left dlPFC using the modified Beam F3 method, as is commonly done in clinical treatments for major depressive disorder [21]. Intermittent theta-burst stimulation (iTBS) was triplet 50 Hz bursts, repeated at 5 Hz; 2 seconds on and 8 s off; 1800 pulses per session; with a total duration of approximately 9.5 minutes. The treatment target intensity was at 110% resting motor threshold determined using a standard visual observation method of the fingers of the right hand. We chose this intensity of stimulation dose with the intent of balancing tolerability and effectiveness. Based on a past study examining the necessity of increased stimulation intensity for older adults due to prefrontal atrophy [22], we felt that stimulating at 110% MT was reasonable especially given that the SAINT trials [16, 18] treated at 90% MT adjusted for differences in distance from coil-cortex over the motor cortex and dl-PFC. Knowing that our sample would not primarily consist of older adults, we decided that 110% MT would be sufficient to stimulate the cortex. Treatment intensity was adjusted for tolerability with titrating to the target dose as soon as possible.

#### 2.4.2. Treatment 2B Parameters (Accel-TMS iTBS+10 Hz dmPFC)

Stimulation was delivered with the active side of the cool D-B80 A/P coil positioned over the midline (bilateral) dmPFC using 25.8% of the distance from nasion to inion. iTBS was triplet 50 Hz bursts, repeated at 5 Hz; 2 seconds on and 8 seconds off; 600 pulses per session; duration approximately 3 minutes and 10 seconds. Immediately following iTBS, 10 Hz TMS was 4 s on and 11 s off; 1200 pulses per session; duration approximately 7 minutes and 30 seconds. The total protocol of iTBS+10 Hz was 1800 pulses per session, taking approximately 10 minutes and 40 seconds. Given the lack of clear parameters for treating dysphoria with accel-TMS over the dmPFC, the choice of iTBS followed by 10 Hz was based on several pain studies that demonstrated remarkable improvement with these parameters [23, 24]. Additionally, there were concerns regarding the tolerability of iTBS over the dmPFC as higher intensity stimulation is generally needed to reach the target area. The treatment target intensity was at 110% resting motor threshold determined using a standard visual observation method of the foot. Treatment intensity was adjusted for tolerability with titrating to the target dose as soon as possible.

#### 2.4.3. Choice of Protocol

There are many considerations for advancing accel-TMS protocols (e.g., number of sessions per day, anatomical positioning, tolerability, and stimulation frequency). In prior studies, the use of 2 sessions per day did not show benefit, while 10 sessions did [16, 25]. Pragmatically, we selected 5 sessions to better fit patient and staff schedules. Anatomic targeting remains an active area of research for TMS, with clinicians balancing the potential benefits with logistical and cost considerations. Methods may include individual fMRI metrics of brain function [26, 27] or structural MRI to target symptom clusters [28]. We pragmatically chose methods that do not require expensive or complex neuronavigation, starting with the Beam F3 location that has yielded solid efficacy in depression [21, 29]. If insufficient benefit was found, we could quickly switch to an easy-to-localize dmPFC similar to what has been done for OCD [30, 31].

### 2.5. Outcome Measurements

As this was a pilot study, no specific power analysis was performed. The sample size was based on the expected number of participants to demonstrate feasibility and provide some preliminary results regarding effectiveness.

For phase 1, we determined feasibility, tolerability, and preliminary effectiveness of VR by descriptive statistics of enrollment, side effects, participant ratings of likeability, and clinical response and remission. Our primary outcome measure for benefit across the entire sample was the SF-36 short form emotional well-being subscale (SF-36 EWB). We initially planned to use a summation score of the SF-36 scale which captures overall health to assess benefit; however, upon further investigation, we discovered that an overall summation score was not validated for the scale [32]. We then chose a subscale of the SF-36, the SF-36 EWB, that we felt best encompasses the various aspects of dysphoria (e.g., feelings of nervousness or sadness and overall happiness) across all syndromes studied. The primary outcome measure was chosen to differ from the criteria for progression throughout the study to avoid possible rating bias (e.g., scoring SF-36 EWB as worse than in reality to move on to the next phase of the study) impacting results. Our secondary outcome measure for benefit across the entire sample (i.e., regardless of symptom severity at baseline) was the clinician-administered scales that tracked each disorder (e.g., depression = MADRS, anxiety = HAM-A, PTSD = CAPS-5, and pain = clinician administered PROMIS pain interference). Exploratory measures for benefit across the entire sample included the self-rated scales that tracked each disorder (e.g., depression = PHQ-9, anxiety = GAD-7, PTSD = PCL-5, and pain = PROMIS pain intensity). We tested for significant improvement in rating scores from baseline to the VR endpoint (visit 10 or alternate data point dependent on VR exit visit) (paired *t*-test). Significance was defined as *p* < 0.05, two-sided.

To clarify the effect of treatment on participants that had significant symptom severity (i.e., depression, anxiety, PTSD, and chronic pain) related to a diagnosis, we sorted participants into each group based on whether they met eligibility criteria (see above for diagnosis-specific patient rating scale severity) for the disorder. Individuals with comorbid conditions were placed into multiple groups as long as they met the criteria for a particular group. Our primary outcome measure for diagnosis-specific outcome was the clinician-administered scale that tracked each disorder. Our secondary outcome measure was the self-rated scale that tracked each disorder. We tested for significantly greater improvement in rating scores from baseline to the VR endpoint (paired *t*-test). Significance was defined as *p* < 0.05, two-sided.

For phase 2, we assessed tolerability by side effect profile and the average percent of resting motor threshold (MT) reached compared to the target of 110% MT by the end of treatment for each stimulation parameter. We determined feasibility and effectiveness by descriptive statistics of the number enrolled, number completed, and clinical response and remission. For benefit across the entire sample in phase 2A, our primary outcome measure was the SF-36 EWB subscale. Our secondary outcome measure for benefit across the entire sample (i.e., regardless of symptom severity at baseline) was the clinician-administered scales that tracked each disorder. Exploratory measures included the self-rated scales that tracked each disorder. We tested for significant improvement in rating scores from treatment A1 to follow-up A1 (paired *t*-test). Significance was defined as *p* < 0.05, two-sided.

For benefit within specific diagnostic groups in phase 2A, we followed the same procedure as phase 1 (see above). Our primary outcome measure within diagnostic groups was the clinician-administered scale that tracked each disorder. Our secondary outcome measure was the self-rated scale that tracked each disorder. We tested for significant improvement in rating scores from “treatment A1” to “follow-up A1” (paired *t*-test). Significance was defined as *p* < 0.05, two-sided.

To analyze sustained clinical improvement, we recorded rating scores from “follow-up A1” and “follow-up A5” and examined for sustained clinical response and remission. A meaningful change in SF-36 EWB scores was defined as ≥30% increase in SF-36 EWB subscores. Additionally, the clinical response and remission definition for each disorder were as follows. For depression: response = ≥50% decrease in MADRS score; remission = score ≤ 10 MADRS. For anxiety: response = ≥50% decrease in HAM-A score; remission = score ≤ 7 HAM-A. For PTSD: response = ≥30% decrease in CAPS-5 score; remission = no diagnosis of PTSD based on CAPS-5 criteria. For chronic pain: response = ≥30% decrease in PROMIS pain interference score; remission = ≥50% decrease in PROMIS pain interference score.

To determine the benefit in those who went on to phase 2B because they did not benefit from phase 2A, we repeated the statistical measures completed on phase 2A for phase 2B (i.e., feasibility and tolerability, paired *t*-test analysis across the entire sample, paired *t*-test analysis within diagnostic groups, and sustained response and remission).

## 3. Results

**Figure 2:**
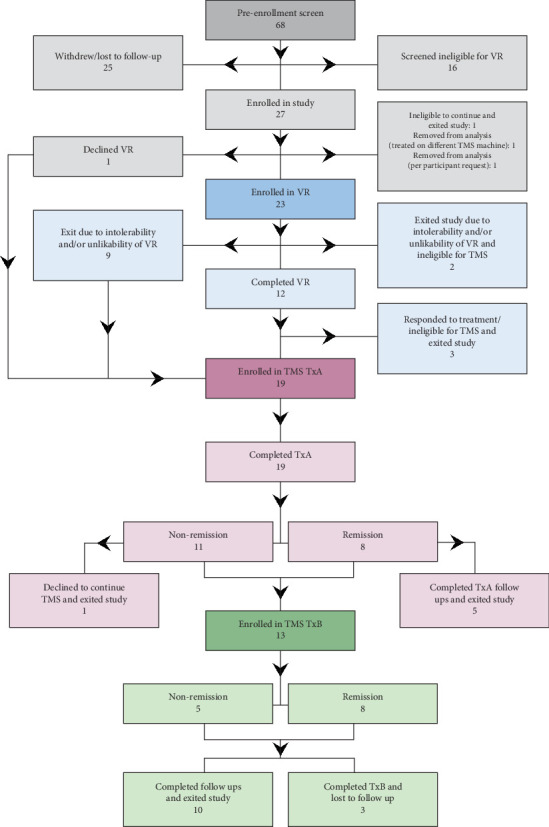
Participant flow throughout the study.

There were 24 participants enrolled in the study (Figure 2). Of those participants, the mean age was 43.6 years (SD = 15.8). Additional demographics are described in Table 2. At the beginning of participation (i.e., regardless of entry phase), 19 qualified for depression, 17 qualified for anxiety, 10 qualified for PTSD, and 17 qualified for chronic pain based on study eligibility criteria. Of the 20 participants analyzed in phase 1, 14 qualified for depression, 15 qualified for anxiety, 8 qualified for PTSD, and 13 qualified for chronic pain. Of the 19 participants analyzed in phase 2A, 15 qualified for depression, 16 qualified for anxiety, 8 qualified for PTSD, and 12 qualified for chronic pain. Of the 12 participants analyzed in phase 2B, 8 qualified for depression, 10 qualified for anxiety, 3 qualified for PTSD, and 9 qualified for chronic pain. Assessing the degree of comorbidity for the total sample of 24 participants, 9 met symptom severity for all four diagnoses, 2 met symptom severity for three diagnoses, 8 met symptom severity for two diagnoses, and 5 met symptom severity for one diagnosis.

### 3.1. Acute Change of the Entire Sample for Each Phase (VR Mindfulness, Accel-TMS Left dlPFC, and Accel-TMS dmPFC)

One participant was excluded from phase 1 primary analysis, and three were excluded from all phase 1 analyses due to incomplete data. We completed pairwise *t*-tests to assess acute change in each phase. Across the entire sample in phase 1: VR mindfulness, there was no significant change in the primary measure of the SF-36 EWB (*n* = 19, *t*(18) = −1.253, *p* = .226) from VR visit 1 to the designated VR endpoint (last day of VR or first day of TMS). However, we found a significant change in one of the secondary measures, the CAPS-5 (*n* = 20, *t*(19) = 2.357, *p* = .029), as well as two exploratory measures (*n* = 20) (PHQ-9, *t*(19) = 2.794, *p* = .012; PCL-5, *t*(19) = 3.319, *p* = .004). All other secondary and exploratory measures were not significant (Table 3).

In phase 2A: accel-TMS left dlPFC, we found significant change from treatment A1 to follow-up A1 in the primary measure of the SF-36 EWB (*n* = 19, *t*(18) = −2.246, *p* = .038) across the entire sample. The secondary measures for depression, anxiety, and PTSD demonstrated significant change as well (*n* = 19) (MADRS, *t*(18) = 2.852, *p* = .011; HAM-A, *t*(18) = 3.234, *p* = .005; CAPS-5, *t*(18) = 2.841, *p* = .011). Additionally, all exploratory measures for depression, anxiety, PTSD, and chronic pain significantly changed (*n* = 19) (PHQ-9, *t*(18) = 2.647, *p* = .016; GAD-7, *t*(18) = 3.586, *p* = .002; PCL-5, *t*(18) = 3.353, *p* = .004; PROMIS pain intensity, *t*(18) = 2.282, *p* = .035). Only one secondary measure, PROMIS pain interference, was not significant (Table 4).

One participant was removed from all phase 2B analyses due to incomplete data. In phase 2B: accel-TMS dmPFC, there was no significant change in the primary measure of the SF-36 EWB from treatment B1 to follow-up B1 (*n* = 12, *t*(11) = −1.864, *p* = .089). However, secondary measures of depression and anxiety significantly changed (*n* = 12) (MADRS, *t*(11) = 3.113, *p* = .010; HAM-A, *t*(11) = 2.603, *p* = .025) as well as exploratory measures of depression, anxiety, and PTSD (*n* = 12) (PHQ-9, *t*(11) = 2.289, *p* = .043; GAD-7, *t*(11) = 2.488, *p* = .030; PCL-5, *t*(11) = 2.257, *p* = .045). All other secondary and exploratory measures were not significant (Table 5).

### 3.2. Acute Change by Diagnostic Group for Each Phase (VR Mindfulness, Accel-TMS Left dlPFC, and Accel-TMS dmPFC)

We completed pairwise *t*-tests to assess acute change within diagnostic groups (e.g., participant met inclusion criteria by self-report scale) in each phase. There was no significant change in the primary clinician-rated measures in any group in phase 1: VR mindfulness from visit 1 to the designated VR endpoint. However, we found significant changes in secondary self-rated measures within the depression (PHQ-9, *n* = 16, *t*(15) = 3.148, *p* = .007) and PTSD groups (PCL-5, *n* = 8, *t*(7) = 3.705, *p* = .008). All other secondary measures were not significant (Table 6). In phase 2A: accel-TMS left dlPFC, we found significant changes in the primary clinician-rated and secondary self-rated measures of the depression (*n* = 15) (MADRS, *t*(14) = 3.608, *p* = .003; PHQ-9, *t*(14) = 2.902, *p* = .012), anxiety (*n* = 16) (HAM-A, *t*(15) = 3.049, *p* = .008; GAD-7, *t*(15) = 3.098, *p* = .007), and PTSD groups (*n* = 8) (CAPS-5, *t*(7) = 2.581, *p* = .036; PCL-5, *t*(7) = 2.689, *p* = .031) from treatment A1 to follow-up A1. We did not find significant change in the pain group for either primary or secondary measures (Table 7). For phase 2B: accel-TMS dmPFC, we found significant changes in primary clinician measures in the depression (MADRS, *n* = 8, *t*(7) = 2.656, *p* = .033) and anxiety groups (HAM-A, *n* = 10, *t*(9) = 2.702, *p* = .024), as well as the secondary self-rated measure in the anxiety group (GAD-7, *n* = 10, *t*(9) = 2.617, *p* = .028) from treatment B1 to follow-up B1. There was no significant change in the PTSD or chronic pain groups (Table 8).

### 3.3. Clinical Response and Remission across Each Phase

**Figure 3:**
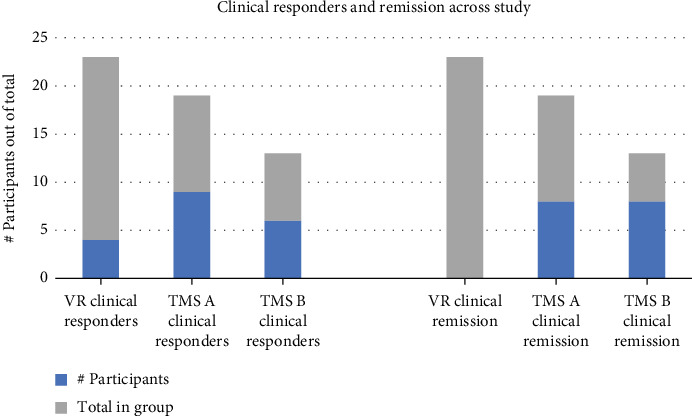
Clinical response and remission across the entire study.

As an exploratory outcome, we examined the number of participants who achieved a clinical response and/or remission in at least one of their conditions for which they met severity criteria (e.g., depression, anxiety, PTSD, and/or chronic pain; response and remission are defined for each condition the same as above) across each phase of the study. In phase 1: VR mindfulness (*n* = 23), 4 showed a clinical response, but none achieved clinical remission by the end of participation in that phase. In phase 2A: accel-TMS left dlPFC (*n* = 19), 9 showed a clinical response, and 8 achieved clinical remission by the end of participation in that phase. In phase 2B: accel-TMS dmPFC (*n* = 13), 6 showed a clinical response, and 8 achieved clinical remission by the end of participation (Figure 3). When looking at individual diagnostic groups from the beginning to the end of phase 2 (treatments A and B), 8 of the 15 who originally presented with depression, 11 of the 16 who originally presented with anxiety, and 7 of the 8 who originally presented with PTSD either achieved a clinical response or remission by the end of accel-TMS treatment.

### 3.4. Accel-TMS Left dlPFC and dmPFC Sustained Improvement

For phase 2A: accel-TMS left dl-PFC follow-up, participants who showed meaningful wellness benefit (≥30% improvement in primary self-rated scale) entered a follow-up phase rather than progress straight to phase 2B. Of those who entered weekly follow-ups, there was no significant change in SF-36 EWB scores from follow-up A1 (Mean = 60.0) to follow-up A5 (Mean = 64.5) (*n* = 8, *t*(7) = −1.116, *p* = .301). Additionally, response and remission rates from baseline were determined from clinician-rated scale scores for follow-up A1 and follow-up A5. The number of participants slightly increased that met the response (4 to 6) and remission (3 to 6) criteria for follow-up A1 to follow-up A5. No diagnostic subgroup had a decline in response or remission rates by the end of phase 2A (Table 9).

In phase 2B: accel-TMS dmPFC follow-up, all participants were offered follow-up visits regardless of wellness benefit. Of those who completed weekly phase 2B follow-ups, there was no significant change in SF-36 EWB scores from follow-up B1 (Mean = 52.0) to follow-up B5 (Mean = 47.56) (*n* = 9, *t*(8) = .912, *p* = .388). Additionally, response and remission rates from baseline were determined from clinician-rated scale scores for follow-up B1 and follow-up B5. The number of participants showing a response (5 to 5) remained stable from follow-up B1 to follow-up B5, and those meeting remission (6 to 5) dropped slightly (Table 9).

### 3.5. Tolerability and Feasibility of Treatments

**Figure 4:**
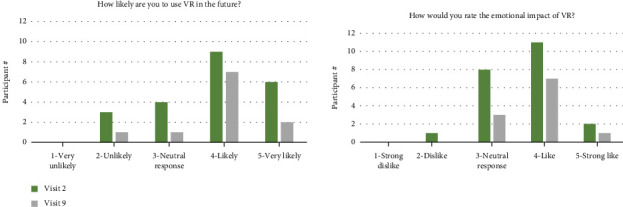
Participant ratings of VR likelihood and emotional impact.

Side effects experienced during VR included fatigue during sessions, heightened emotions, nausea, and one instance of motion sickness and perceived possible worsening of symptoms. Only one individual stopped VR due to side effects (e.g., motion sickness and nausea), six stopped due to perceived lack of benefit, and four stopped for other reasons (Table 10). Of the 23 participants who started VR, twelve completed all 10 visits, six completed between 5 and 9 visits, and five completed less than 5 visits. On a user experience survey completed at the beginning and end of VR treatment (visits 2 and 9), most participants stated that they would be likely to use VR in the future to manage their symptoms and had a positive rating of the emotional impact of VR (Figure 4).

For left dlPFC accel-TMS, side effects included mild headache, slight nausea, pain/tenderness during stimulation, tiredness, mild scalp soreness, fatigue, mild eyebrow sensations after treatment, and one instance of vomiting due to anxiety, and perception that the individual's face was asymmetrical (not observable by staff). No participants stopped treatment due to side effects or other reasons. In dmPFC accel-TMS, side effects included mild headache, tiredness, insomnia, and one instance of minor brief disorientation, positive feelings, and pain/tenderness during stimulation. No participants stopped treatment due to side effects, and four discontinued follow-up sessions for other reasons (Table 10).

**Figure 5:**
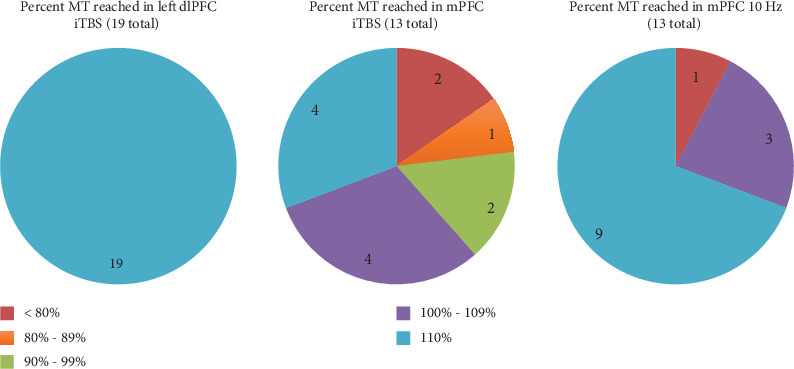
Percent MT reached during each accel-TMS protocol.

All participants receiving left dlPFC accel-TMS iTBS (*n* = 19) were able to reach their target treatment at 110% of their MT by the end of phase 2A. For those who continued to phase 2B (*n* = 13), dmPFC accel-TMS was less tolerable during the iTBS protocol than the 10 Hz protocol. During dmPFC iTBS, a little more than half (*n* = 8) of participants reached between 100% and 110% of their MT, and the remainder (*n* = 5) were unable to reach at least 100% by the end of phase 2B. During dmPFC 10 Hz stimulation, most (*n* = 9) of the participants reached 110% of their MT, and the remainder (*n* = 4) reached less than 110% (Figure 5). On average, for those who moved on to phase 2B, their phase 2B foot MT was higher than their phase 2A hand MT (phase 2A: Avg MT = 46.92% machine output, SD = 11.11; phase 2B: Avg MT = 58.77% machine output, SD = 10.78).

## 4. Discussion

This pilot stepwise approach to treat participants with transdiagnostic dysphoria shows promise, especially in the speed of trialing multiple approaches that can have strong efficacy for an individual. We sought to explore how sequencing two evidence-based treatments, mindfulness and TMS, can be implemented in a novel way. To do this, we used emergent technology (i.e., VR-delivered mindfulness) and science (i.e., innovative accel-TMS protocols with varied anatomical targeting). We demonstrated the feasibility, acceptability, and potential effectiveness in a diagnostically broad population with multiple comorbidities (reflecting clinical practice). To enhance our understanding and improve future approaches, we expect that conducting studies on participant selection, identifying the best outcome measurements for individuals with dysphoria, and selecting appropriate TMS parameters will be valuable.

### 4.1. VR Mindfulness in a Transdiagnostic Sample of Dysphoria

#### 4.1.1. Feasibility and Acceptability of VR

VR mindfulness did not induce adverse effects and was generally pleasing to participants; however, a notable number became frustrated with the lack of treatment effects. Mindfulness training delivered in a media format can increase scalability over in-person training, and VR uniquely focuses attention away from environmental distractions. Although only a few individuals experienced minor side effects while using VR, some people may not tolerate wearing a headset for an immersive experience. Therefore, to broaden the utilization of VR, monitoring future advancements in VR technology or considering alternative media formats that can be used in a calm environment may be beneficial. VR served a useful purpose in this study and has implications for future research and clinical implementation as it could result in a cost-effective and scalable first-line treatment approach, especially with respect to integrating elements of regular clinical visits that may effectively alleviate symptoms due to nonspecific factors (e.g., interaction with friendly staff and consistent positive routine). Additionally, in research, the lead-in portion of VR can help with the enrichment of the sample in that full and partial responders can be identified prior to further treatment and time investment.

#### 4.1.2. Effectiveness and Outcomes of VR

Effectiveness of VR mindfulness was limited for our treatment-resistant population. Many of our participants had lengthy histories of illness and treatments trialed, so many had prior exposure to components of mindfulness. Nonetheless, some noted perceived benefit or interest in continuing to use VR to manage symptoms, and there was significant change in participant and clinician ratings of PTSD symptoms. VR technology has been employed in the treatment of anxiety and PTSD, so VR mindfulness may be effective as a first-line intervention before symptoms begin to escalate [9]. From a research perspective on procedural therapies, VR mindfulness may be a useful filter for participants who may respond to simple interventions, may help establish the stability of symptoms as a run-in period, and may facilitate reliable engagement in the subsequent procedural phase.

### 4.2. Accelerated TMS in a Transdiagnostic Sample of Dysphoria

#### 4.2.1. Feasibility and Acceptability of Accel-TMS

Accel-TMS was generally well-tolerated, and the side effects reported were consistent with standard TMS treatment [33, 34]. We found that the speed of anticipated response aided in rapidly switching to the next treatment within a manageable timeframe when a participant did not respond to initial intervention. Additionally, we found that the 5 sessions/day for a 5-day schedule was preferable for some participants to better fit their schedules. A notable exploratory finding was that dmPFC iTBS was more painful than dmPFC 10 Hz stimulation, to the point of limiting the full power of stimulation. Although iTBS has gained prominence with accelerated protocols, 10 Hz or other frequencies may be reasonable alternatives. While there are many possibilities for optimizing accel-TMS approaches, practical considerations can help guide various protocol selections (e.g., number of sessions per day, anatomical positioning, tolerability, and stimulation frequency).

#### 4.2.2. Effectiveness and Outcomes of Accel-TMS

Accel-TMS provided significant improvement in symptoms of several conditions during treatment as well as a positive impact on emotional well-being. There were clear, meaningful benefits as participants progressed through each phase of the study. Many of those that did not respond to the initial VR intervention did respond to left dlPFC accel-TMS, and participants continued to improve through dmPFC accel-TMS. Our findings are consistent with the current body of research regarding the effectiveness of accel-TMS in transdiagnostic populations [34]. Additionally, our results provide preliminary support that continuing TMS using a different location and/or protocol can be effective for patients who do not respond to initial intervention. Similar studies support the 5 treatments/day for a 5-day schedule in participants with depression [17], and our findings are consistent with other studies on the effect of standard TMS on anxiety and PTSD [11, 35–37]. However, there is still a significant need to optimize and establish accelerated TMS for clinical implementation.

We had a small sample size for several of our groups, most notably those who met the criteria for PTSD during dmPFC accel-TMS. Paired *t*-test analysis did not find a significant change within that group; however, it was likely due to sample size as the group size included only 3 individuals, all of whom met clinical remission by the time they completed phase 2B. Additionally, there was no clear evidence of chronic pain improvements in our sample. This does not align with current research, as TMS has been shown to have a clinically sound effect on chronic pain [10]. This may have been due to patient selection, measures used to assess outcome, or timeframe of assessment.

### 4.3. Limitations and Future Direction

This was a pilot study with a variable participant population reflective of clinical reality. Research must advance broad conceptual approaches to tracking and addressing emotional well-being across complex and comorbid phenotypes. Although the SF-36 as a whole can be helpful in assessing overall health, a different scale may provide a better picture of emotional well-being in the future. This study provided a signal that similar protocols may be able to be used to treat a broader population; however, efficacy in relationship to protocol selections is an important area for future research. Exploration within individual groups will likely be needed, as well as varying the order of protocols (e.g., accel-TMS then VR, both at the same time, or TMS followed by VR). Modifications in participant selection and other protocol variations, including changing the order of VR to accel-TMS, could enhance the stepwise progression from VR to accelerated TMS or indicate the potential integration of these interventions with other therapies. The sample size of this pilot study is small, and thus, some of the nonsignificant results may be due to low power. Future studies with larger samples are needed to more robustly test the effectiveness of the protocols.

Another limitation is our study design, which provided unblinded active treatment to all participants in a stepwise manner. There was no randomized sham-controlled arm, so inferences about placebo responding can only be drawn based on prior treatment failure. Further work is needed with blinded sham-controlled randomized samples in order to determine if response and remission are dependent on anatomic location versus extending treatment at the same brain site from 1 to 2 weeks. Further, single TMS treatments were given once weekly for six weeks to those who experienced benefit from left dlPFC accel-TMS by one week following treatment. Those without benefit quickly moved on to dmPFC accel-TMS. Given the timeframe from completing the dlPFC accel-TMS treatment and starting the dmPFC accel-TMS, delayed effects from the dlPFC accel-TMS may have played a role on the impact of dmPFC stimulation effectiveness. The impact of delayed effects requires further investigation [19]. While we saw rapid responses, we also need to demonstrate durability of response with long-term follow-up studies.

Inclusion criteria to better address a population need (e.g., selection based on pain phenotype, severity, or pain interference) may better evaluate the effect of accel-TMS on chronic pain. Furthermore, pain intensity has been reported as a lagging indicator of patient improvement, so our data capture may not have extended long enough to detect an effect [38].

## 5. Conclusions

This stepwise approach provides a path for addressing symptoms of dysphoria, with effective options for those who do not experience symptom improvement with the initial intervention. Accelerated TMS shows great potential in treating transdiagnostic depression, anxiety, and PTSD. Modifications in participant selection and other protocol variations could enhance the stepwise progression from VR to accelerated TMS or indicate the potential integration of these interventions with other therapies.

## Figures and Tables

**Table 1:** Clinician- and self-rated scales completed throughout the study.

Assessments	VR visit 1	VR visit 5	VR visit 10	TMS TxA1	TMS TxA5	TMS FxA1–A5	TMS TxB1	TMS TxB5	TMS FxB1–B5
**Demographics**	X			X			X		
**SF-36**	X	X	X	X	X	X	X	X	X
**PHQ-9**	X	X	X	X	X	X	X	X	X
**GAD-7**	X	X	X	X	X	X	X	X	X
**PCL-5**	X	X	X	X	X	X	X	X	X
**LEC-5**	X			X			X		
**PROMIS pain intensity**	X	X	X	X	X	X	X	X	X
**PROMIS sleep**	X	X	X	X	X	X	X	X	X
**PROMIS fatigue**	X	X	X	X	X	X	X	X	X
**DARS**	X	X	X	X	X	X	X	X	X
**POMS**	X	X	X	X	X	X	X	X	X
**SETS**	X			X			X		
**Stanford hypersensitivity scale**	X		X						
**MAIA**	X		X						
**User experience**	X		X						
**TMS safety screen**			X	X			X		
*MADRS*	X		X	X		X	X		X
*HAM-A*	X		X	X		X	X		X
*CAPS-5*	X		X	X		X	X		X
*PROMIS pain interference*	X		X	X		X	X		X
*YMRS*	X		X	X		X	X		X
*C-SSRS*	X		X	X		X	X		X
*Medical history*	X			X			X		
*Clinical interview*	X		X	X		X	X		X
Urine drug and pregnancy screen				X					

Bold indicates self-rated scales, and italic indicates clinician-rated scales. SF-36 = 36-item short form survey; PHQ-9 = patient health questionnaire-9; GAD-7 = generalized anxiety disorder-7; PCL-5 = PTSD checklist for DSM-5; LEC-5 = life events checklist for DSM-5; PROMIS = patient-reported outcomes measurement information system; DARS = dimensional anhedonia rating scale; POMS = profile of mood states; SETS = Stanford expectations of treatment scale; MAIA = multidimensional assessment of interoceptive awareness; MADRS = Montgomery-Asperg depression rating scale; HAM-A = Hamilton anxiety rating scale; CAPS-5 = clinician-administered PTSD scale for DSM-5; YMRS = Young mania rating scale; C-SSRS = Columbia suicide severity rating scale).

**Table 2:** Demographics of study sample.

Variables	*N*	Percent	Mean	SD
Age	22	92%	43.6	15.8
Undisclosed⁣^∗^	2	8%		
Biological sex				
Male	9	38%		
Female	15	63%		
Other	0	0%		
Marital status				
Single	8	33%		
Married	12	50%		
Divorced	0	0%		
Widowed	2	8%		
Domestic partner	0	0%		
Significant other	2	8%		
Race				
American Indian or Alaska Native	0	0%		
Asian	1	4%		
Black or African-American	0	0%		
Native Hawaiian or Pacific Islander	0	0%		
White	22	92%		
More than one race	0	0%		
Unknown or not reported	1	4%		
Ethnicity				
Hispanic or Latino	1	4%		
Not Hispanic or Latino	21	88%		
Unknown or not reported	2	8%		
Veteran	1	4%		
Education				
Less than high school diploma	0	0%		
High school graduate/GED	1	4%		
Some college but no degree	4	17%		
Associate degree	2	8%		
Bachelor's degree	7	29%		
Master's degree	6	25%		
Ph.D., J.D., or other professional degree	4	17%		
Household income				
$0-25,000	1	4%		
$25,001-50,000	5	21%		
$50,001-75,000	7	29%		
$75,001-100,000	4	17%		
$100,001-150,000	2	8%		
$150,001-200,000	3	13%		
$200,001+	1	4%		
Undisclosed	1	4%		
Handedness				
Left	3	12%		
Ambidextrous	0	0%		
Right	21	88%		

⁣^∗^Although participants did not indicate age, both were clearly adults greater than 18 years old.

**Table 3:** Paired *t*-tests of primary clinician and self-rated scales from VR visit 1 to the designated VR Endpoint across the entire sample.

Measure	Mean		Std. error mean difference	Mean difference	*t*	df	Sig. (2-tailed)
	VR visit 1	VR endpoint					
PHQ-9	15.40	13.85	.5548	1.5500	2.794	19	.012
GAD-7	12.80	12.40	.8284	.4000	.483	19	.635
PCL-5	32.70	26.30	1.9282	6.4000	3.319	19	.004
PROMIS pain intensity	5.00	5.25	.2702	-.2500	-.925	19	.367
SF-36 emotional well-being	33.47	37.47	3.1936	-4.000	-1.253	18	.226
MADRS	28.80	26.55	1.2935	2.2500	1.740	19	.098
HAM-A	23.05	21.05	1.2031	2.0000	1.662	19	.113
CAPS-5	30.30	26.05	1.8033	4.2500	2.357	19	.029
PROMIS pain interference	12.55	12.15	.7588	.4000	.527	19	.604

**Table 4:** Paired *t*-tests of primary clinician and self-rated scales from TMS treatment A1 to TMS follow-up A1 across the entire sample.

Measure	Mean		Std. error mean difference	Mean difference	*t*	df	Sig. (2-tailed)
	TxA1	FxA1					
PHQ-9	15.42	11.68	1.4118	3.7368	2.647	18	.016
GAD-7	14.00	9.05	1.3796	4.9474	3.586	18	.002
PCL-5	32.68	18.95	4.0967	13.7368	3.353	18	.004
PROMIS pain intensity	4.89	4.42	.2076	.4737	2.282	18	.035
SF-36 emotional well-being	34.32	43.79	4.2187	-9.4737	-2.246	18	.038
MADRS	28.68	20.84	2.7500	7.8421	2.852	18	.011
HAM-A	22.63	16.00	2.0508	6.6316	3.234	18	.005
CAPS-5	26.89	18.68	2.8904	8.2105	2.841	18	.011
PROMIS pain interference	12.47	12.47	.5407	.0000	.000	18	1.000

**Table 5:** Paired *t*-tests of primary clinician and self-rated scales from TMS treatment B1 to TMS follow-up B1 across the entire sample.

Measure	Mean		Std. error mean difference	Mean difference	*t*	df	Sig. (2-tailed)
	TxB1	FxB1					
PHQ-9	13.92	10.17	1.6382	3.7500	2.289	11	.043
GAD-7	11.58	8.17	1.3732	3.4167	2.488	11	.030
PCL-5	21.58	15.50	2.6953	6.0833	2.257	11	.045
PROMIS pain intensity	4.75	4.58	.2973	.1667	.561	11	.586
SF-36 emotional well-being	39.67	48.67	4.8273	-9.0000	-1.864	11	.089
MADRS	22.25	14.50	2.4898	7.7500	3.113	11	.010
HAM-A	17.33	9.58	2.9775	7.7500	2.603	11	.025
CAPS-5	17.75	11.58	2.9869	6.1667	2.065	11	.063
PROMIS pain interference	11.50	11.58	.9411	-.0833	-.089	11	.931

**Table 6:** Paired *t*-tests of primary clinician and self-rated scales from VR visit 1 to the designated VR endpoint within diagnostic groups.

Measure	Mean		Std. error mean difference	Mean difference	*t*	df	Sig. (2-tailed)
	VR visit 1	VR endpoint					
Depression							
PHQ-9	17.25	15.31	.6156	1.938	3.148	15	.007
MADRS	31.25	29.00	1.4986	2.2500	1.501	15	.154
Anxiety							
GAD-7	15.33	13.93	.8826	1.4000	1.586	14	.135
HAM-A	26.13	23.33	1.3565	2.8000	2.064	14	.058
PTSD							
PCL-5	56.62	47.00	2.5977	9.6250	3.705	7	.008
CAPS-5	50.25	46.50	2.2019	3.7500	1.703	7	.132
Chronic pain							
PROMIS pain intensity	5.87	6.07	.3409	-.2000	-.587	14	.567
PROMIS pain interference	14.73	13.80	.9333	.9333	1.000	14	.334

**Table 7:** Paired *t*-tests of primary clinician and self-rated scales from TMS treatment A1 to TMS follow-up A1 within diagnostic groups.

Measure	Mean		Std. error mean difference	Mean difference	*t*	df	Sig. (2-tailed)
	TxA1	FxA1					
Depression							
PHQ-9	17.53	12.93	1.5851	4.6000	2.902	14	.012
MADRS	31.67	22.00	2.6791	9.6667	3.608	14	.003
Anxiety							
GAD-7	15.50	10.44	1.6342	5.0625	3.098	15	.007
HAM-A	24.19	16.94	2.3779	7.2500	3.049	15	.008
PTSD							
PCL-5	55.38	32.63	8.4595	22.7500	2.689	7	.031
CAPS-5	46.13	30.75	5.9580	15.3750	2.581	7	.036
Chronic pain							
PROMIS pain intensity	6.50	5.83	.3097	.6667	2.152	11	.054
PROMIS pain interference	16.17	16.25	.8657	-.0833	-.096	11	.925

**Table 8:** Paired *t*-tests of primary clinician and self-rated scales from TMS treatment B1 to TMS follow-up B1 within diagnostic groups.

Measure	Mean		Std. error mean difference	Mean difference	*t*	df	Sig. (2-tailed)
	TxB1	FxB1					
Depression							
PHQ-9	17.63	12.50	2.2947	5.1250	2.233	7	.061
MADRS	28.13	18.75	3.5302	9.3750	2.656	7	.033
Anxiety							
GAD-7	13.90	9.80	1.5667	4.1000	2.617	9	.028
HAM-A	19.50	10.30	3.4052	9.2000	2.702	9	.024
PTSD							
PCL-5	49.33	33.33	7.0000	16.0000	2.286	2	.150
CAPS-5	39.67	25.00	7.3106	14.6667	2.006	2	.183
Chronic pain							
PROMIS pain intensity	5.56	5.33	.4006	.2222	.555	8	.594
PROMIS pain interference	13.33	13.44	1.2742	-.1111	-.087	8	.933

**Table 9:** Response and remission within each diagnostic group (i.e., participant met inclusion criteria by self-report scale) for follow-ups A1 and A5 (left dlPFC accel-TMS) and follow-ups B1 and B5 (dmPFC accel-TMS).

	FxA1		FxA5		FxB1		FxB5	
	Response	Remission	Response	Remission	Response	Remission	Response	Remission
Depression	2/6	1/6	5/6	4/6	2/5	1/5	1/5	1/5
Anxiety	4/7	3/7	4/7	4/7	5/8	3/8	4/8	2/8
PTSD	2/3	1/3	2/3	1/3	1/2	2/2	1/2	2/2
Chronic pain	0/3	0/3	3/3	0/3	0/7	0/7	2/7	0/7

**Table 10:** Side effects experienced in VR-guided meditation and accel-TMS.

Virtual reality (23 participants)		
*Side effect*	*# of participants*	*# of incidences*
Fatigue during session	3	3
Heightened emotions	2	2
Nausea	2	2
Motion sickness	1	1
Perceived possible worsening of symptoms	1	1
*Reason for discontinuing early*	*# of participants*	
Stopped due to perceived lack of benefit	6	
Stopped for other reasons	4	
Stopped due to side effects	1	

TMS treatment A (19 participants)		
*Side effect*	*# of participants*	*# of incidences*
Mild headache	4	11
Slight nausea	4	11
Pain/tenderness during stimulation	3	5
Tiredness	3	4
Mild scalp soreness	2	2
Fatigue	1	4
Mild sensation in eyebrow after treatment	1	3
Vomiting	1	1
Perception that face was asymmetrical, not observed by staff	1	1
Stopped due to side effects	0	

TMS treatment B (13 participants)		
*Side effect*	*# of participants*	*# of incidences*
Mild headache	4	6
Tiredness	2	2
Insomnia	1	2
Minor disorientation	1	1
Positive feelings	1	1
Pain/tenderness during stimulation	1	1
*Reason for discontinuing follow-ups*	*# of participants*	
Stopped for other reasons	4	
Stopped due to side effects	0	

## Data Availability

Those interested may contact the principal investigator for details on methods and requirements of data sharing.
